# Antibacterial, Synergistic, and Antibiofilm Activities of *Liquidambar orientalis* Mill. Essential Oil Against Clinically Relevant Bacterial Pathogens

**DOI:** 10.3390/antibiotics15090851

**Published:** 2026-08-31

**Authors:** Ziya İlhan, Reyda Şıklaroğlu, Murad Gürses, Murat Bayezit, Taha Gürsoy, Yavuz Musabeşeoğlu, Özgür Erkan, Şefika Musabeşeoğlu, Mehmet Karaca, Hasan Altan Akkan

**Affiliations:** 1 Department of Microbiology, Faculty of Veterinary Medicine, Balıkesir University, 10145 Balıkesir, Türkiye; 2 Department of Food, Agriculture and Livestock Vocational School, Burdur Mehmet Akif Ersoy University, 15030 Burdur, Türkiye; rkiyici@mehmetakif.edu.tr; 3 Department of Genetics, Faculty of Veterinary Medicine, Balıkesir University, 10145 Balıkesir, Türkiye; mgurses@balikesir.edu.tr; 4 Department of Pharmacology, Faculty of Veterinary Medicine, Burdur Mehmet Akif Ersoy University, 15030 Burdur, Türkiye; muratbayezit@mehmetakif.edu.tr; 5 Agriculture, Livestock and Food Research Application and Research Center, Burdur Mehmet Akif Ersoy University, 15030 Burdur, Türkiye; tgursoy@mehmetakif.edu.tr; 6 Department of Obstetrics and Gynecology, Faculty of Veterinary Medicine, Burdur Mehmet Akif Ersoy University, 15030 Burdur, Türkiye; ymusabeseoglu@mehmetakif.edu.tr; 7 Department of Internal Medicine, Faculty of Veterinary Medicine, Burdur Mehmet Akif Ersoy University, 15030 Burdur, Türkiye; 2240307004@ogr.mehmetakif.edu.tr (Ö.E.); mkaraca@mehmetakif.edu.tr (M.K.); hasanaltanakkan@mehmetakif.edu.tr (H.A.A.); 8 Department of Biochemistry, Faculty of Veterinary Medicine, Burdur Mehmet Akif Ersoy University, Institute of Health Sciences, 15030 Burdur, Türkiye; 2140316003@mehmetakif.edu.tr

**Keywords:** *L. orientalis* Mill. essential oil, GC-MS analysis, antibacterial activity, synergistic interactions, antibiofilm activity

## Abstract

**Background/Objectives:** The increasing prevalence of antimicrobial resistance has reduced the effectiveness of conventional antibiotics, highlighting the need for alternative or adjunct antimicrobial agents. This study aimed to investigate the antibacterial, synergistic, and antibiofilm activities of *Liquidambar orientalis* Mill. essential oil against clinically relevant bacterial pathogens, using 24 bacterial strains representing 12 bacterial species. **Methods:** The volatile constituents of *L. orientalis* essential oil obtained by hydrodistillation of storax balsam were characterized by gas chromatography-mass spectrometry (GC-MS) and gas chromatography-flame ionization detection (GC-FID). Antibacterial activity was evaluated using minimum inhibitory concentration (MIC), minimum bactericidal concentration (MBC), disk diffusion, and agar well diffusion assays against a panel of Gram-positive and Gram-negative bacterial pathogens. Synergistic interactions with erythromycin, amoxicillin, gentamicin, and enrofloxacin were assessed using fractional inhibitory concentration index (FICI) analysis. Antibiofilm activity was also evaluated against representative biofilm-forming strains. **Results:** A total of 90 volatile constituents were identified by GC-MS, of which styrene was the most abundant (68.6%), followed by (*E*)-ethyl cinnamate (6.8%), *α*-pinene (5.9%), and *β*-pinene (2.9%). The essential oil exhibited broad-spectrum antibacterial activity, with an overall inhibition rate of 60.4%. Activity was higher against Gram-positive (77.77%) than Gram-negative (73.33%) bacteria. MIC and MBC values ranged from 15.62 to 250 µg/mL and 31.25 to 250 µg/mL, respectively. FICI analysis revealed strain- and antibiotic-dependent interactions, ranging from synergistic to antagonistic effects. Variable antibiofilm activity was observed, with the strongest inhibition against *Brucella melitensis* and the weakest against *Pseudomonas aeruginosa*. No significant differences in MIC or MBC values were observed between bacterial groups (*p* > 0.05). **Conclusions:** *L. orientalis* essential oil demonstrated in vitro antibacterial activity against several clinically relevant pathogens, with particularly notable activity against *B. melitensis*. The essential oil also showed synergistic interactions with selected antibiotics and variable antibiofilm effects. These findings suggest that *L. orientalis* essential oil may have potential as an antibacterial agent or adjunct to antibiotic therapy; however, further in vivo and clinical studies are required to confirm its therapeutic applicability.

## 1. Introduction

Infectious diseases caused by bacteria continue to pose a significant and growing threat to human and animal health and welfare, while antimicrobial resistance has increasingly complicated their effective treatment [1]. In response to this global challenge, the World Health Organization (WHO) published a global priority list of antibiotic-resistant bacteria in 2017 to guide research and development of new antimicrobial agents. This list includes critical Gram-negative pathogens, such as carbapenem- and third-generation cephalosporin-resistant *Enterobacteriaceae*, including *Escherichia coli* and *Klebsiella pneumoniae*, as well as high-priority Gram-positive bacteria such as methicillin-resistant *Staphylococcus aureus* (MRSA) and vancomycin-resistant *Enterococcus* spp. [2].

The increasing incidence of life-threatening bacterial infections, together with the ability of these pathogens to rapidly develop resistance to existing therapeutic agents, underscores the urgent need for the discovery and development of novel antimicrobial compounds. Ideally, such compounds should exhibit low toxicity, high specificity, and favorable bioavailability. Natural products have long been recognized as a particularly promising source of bioactive molecules with these desirable characteristics [3,4]. Among such sources, *Liquidambar orientalis*, a member of the family *Altingiaceae* and a sweetgum species naturally distributed in southwestern Türkiye as well as adjacent Mediterranean regions, including parts of the Aegean area and Rhodes, represents a plant with promising antibacterial activity. The species has been traditionally used in folk medicine, primarily due to bioactive compounds present in its storax resin and essential oils. Phytochemical studies have shown that extracts obtained from different parts of the plant contain monoterpenes such as *α*-pinene, *β*-pinene, limonene, sabinene, and terpinen-4-ol, as well as resin-derived constituents including cinnamic acid derivatives. Many of these compounds have been reported to exhibit antibacterial activity against only a limited number of bacterial strains, supporting the therapeutic potential of *L. orientalis* while highlighting the need for broader antimicrobial evaluation [5,6,7].

Although several studies have investigated the antibacterial properties of essential oils derived from certain species within the genus *Liquidambar* L. [7,8], to the best of our knowledge, no previous study has evaluated the antibacterial, synergistic, and antibiofilm activities of *L. orientalis* essential oil against a broad panel of clinically relevant bacterial pathogens, including *Brucella melitensis* and other clinically important human and animal isolates. Therefore, the present study aimed to characterize the chemical composition of *L. orientalis* essential oil and to systematically investigate its antibacterial, synergistic, and antibiofilm activities against 24 clinically relevant bacterial isolates.

## 2. Results

### 2.1. GC-MS Analysis of L. orientalis Essential Oil

**Figure 1:**
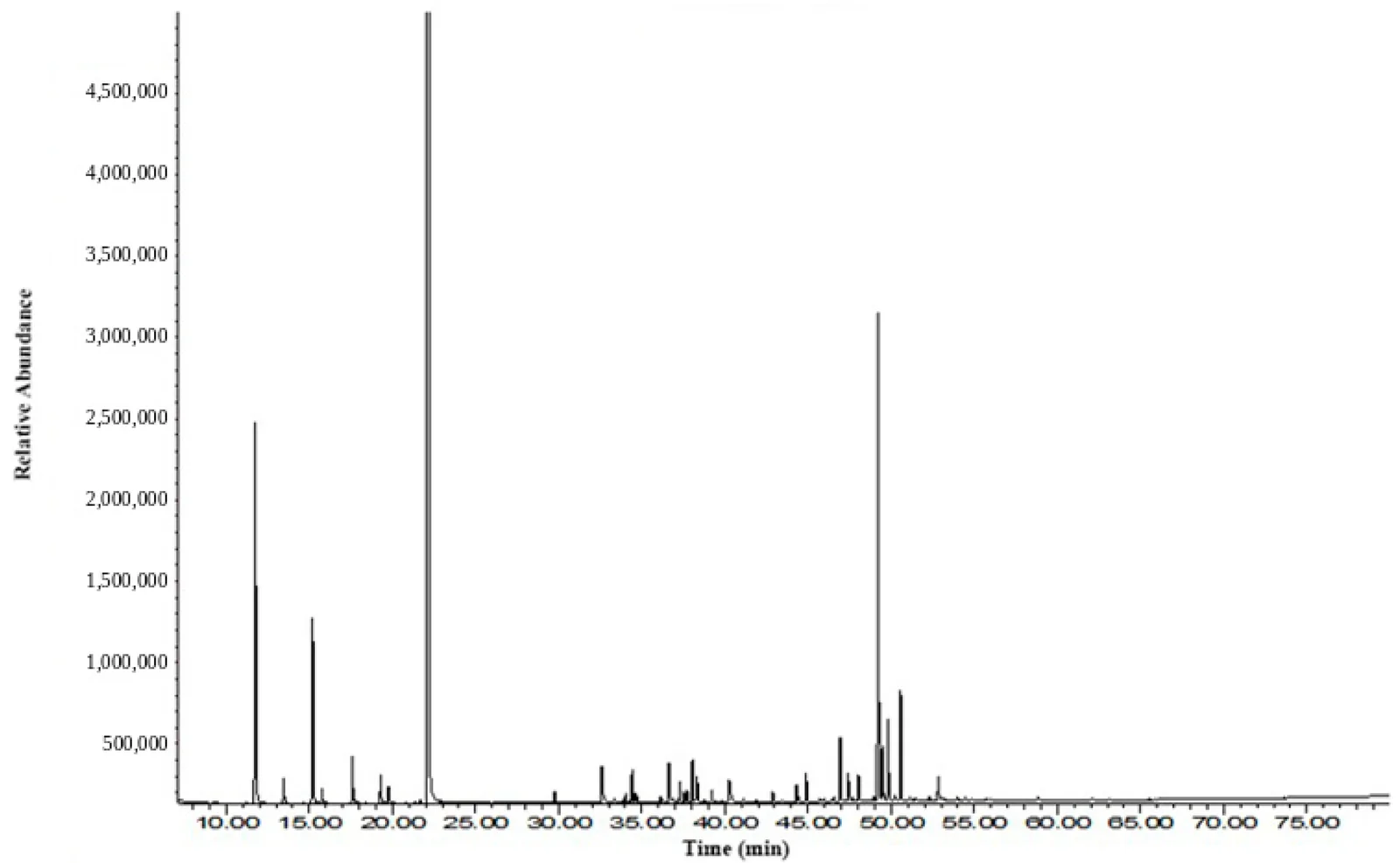
GC-MS chromatogram of *L. orientalis* essential oil. The chromatogram displays the major volatile constituents, with styrene (68.6%), (*E*)-ethyl cinnamate (6.8%) and *α*-pinene (5.9%) identified as the predominant compounds. Other components include low percentages of monoterpenes (*β*-pinene, limonene, *β*-myrcene), sesquiterpenes (*β*-copaene, *γ*-muurolene, *δ*-cadinene) and various aromatic compounds such as 4-ethylphenol and acetophenone.

GC-MS analysis of *L. orientalis* essential oil identified a total of 90 volatile constituents. The monoterpene fraction constituted a major portion of the profile and included *α*-pinene, camphene, *β*-pinene, sabinene, *β*-myrcene, *α*-phellandrene, *α*-terpinene, limonene, *β*-phellandrene and 1,8-cineole, together with minor amounts of (*Z*)-*β*-ocimene, *γ*-terpinene, (*E*)-*β*-ocimene and terpinolene. Aromatic compounds represented the most abundant chemical group, with styrene (68.6%) detected as the predominant constituent, followed by benzaldehyde, methyl chavicol, vinyl phenyl carbinol, 4-ethylphenol and acetophenone. Sesquiterpene hydrocarbons including *α*-cubebene, *β*-cubebene, *β*-copaene, *β*-caryophyllene, allo-aromadendrene, *γ*-muurolene, *α*-muurolene and *δ*-cadinene were present in low to trace quantities. Additionally, several phenylpropanoid derivatives were identified, notably (*E*)-ethyl cinnamate (6.8%), hydrocinnamyl acetate, cinnamyl acetate and cinnamyl alcohol (Table 1, Figure 1).

### 2.2. Antibacterial Activity of L. orientalis Essential Oil

#### 2.2.1. MIC and MBC

**Figure 2:**
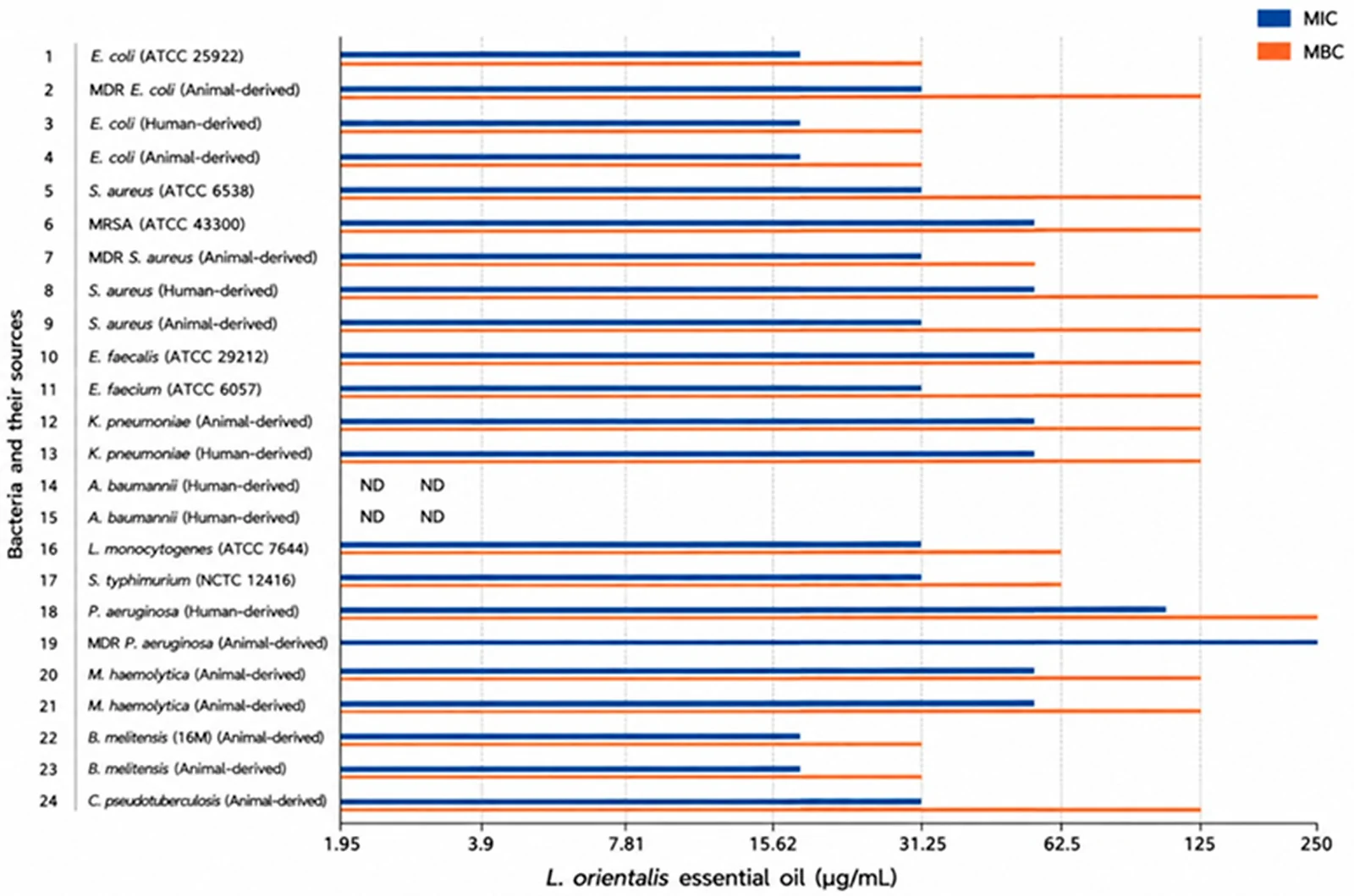
Minimum inhibitory concentration (MIC) and minimum bactericidal concentration (MBC) of *L. orientalis* essential oil against the tested bacterial strains. ND: Not detected.

The essential oil exhibited variable antibacterial activity depending on the bacterial species, with MIC values generally lower than the corresponding MBC values. The MIC, MBC, and MBC/MIC ratios for all tested bacterial isolates are presented in Table 2, while the MIC and MBC values are graphically presented in Figure 2. Based on the MBC/MIC ratios, the essential oil exhibited bactericidal activity against strains with ratios ≤4 and bacteriostatic activity against strains with ratios >4.

#### 2.2.2. Disk Diffusion and Agar Well Diffusion Assays

*L. orientalis* essential oil exhibited antibacterial activity against 58.3% (14/24) and 62.5% (15/24) of the tested bacterial strains, as determined by the disk diffusion and agar well diffusion assays, respectively. The mean antibacterial activity rate across the two diffusion assays was 60.4%. The antibacterial activity was slightly higher against Gram-positive bacteria (77.77%, 7/9) compared with Gram-negative bacteria (73.33%, 11/15). The highest inhibitory effect was observed against *S. aureus*, with an inhibition zone of 14.3 ± 0.1 mm in the disk diffusion assay, and against the *B. melitensis* 16M strain, with an inhibition zone of 16.4 ± 0.2 mm in the agar well diffusion assay (Table 3 and Table 4).

### 2.3. The Fractional Inhibitory Concentration Index (FICI)

Overall, *L. orientalis* essential oil exhibited variable interactions with the tested antibiotics depending on the bacterial strain and antibiotic used, as determined by the fractional inhibitory concentration index (FICI). The interactions ranged from strong synergistic effects (FICI = 0.18–0.41) and additive effects (FICI = 0.66) to indifferent (FICI = 2.76) and antagonistic interactions (FICI = 4.15–8.95) (Table 5).

### 2.4. Antibiofilm Activity Results

The results demonstrated that *L. orientalis* essential oil exhibited varying antibiofilm activities against the tested bacterial strains. The highest and lowest antibiofilm activities among Gram-negative bacteria were observed against animal-derived *B. melitensis* (58.75%) and animal-derived MDR *P. aeruginosa* (18.57%), respectively. Among Gram-positive bacteria, the highest activity was detected against animal-derived *S. aureus* (46.00%), whereas the lowest was observed against *E. faecalis* (ATCC 29212) (27.66%) (Table 6).

### 2.5. Statistical Analysis Results

**Figure 3:**
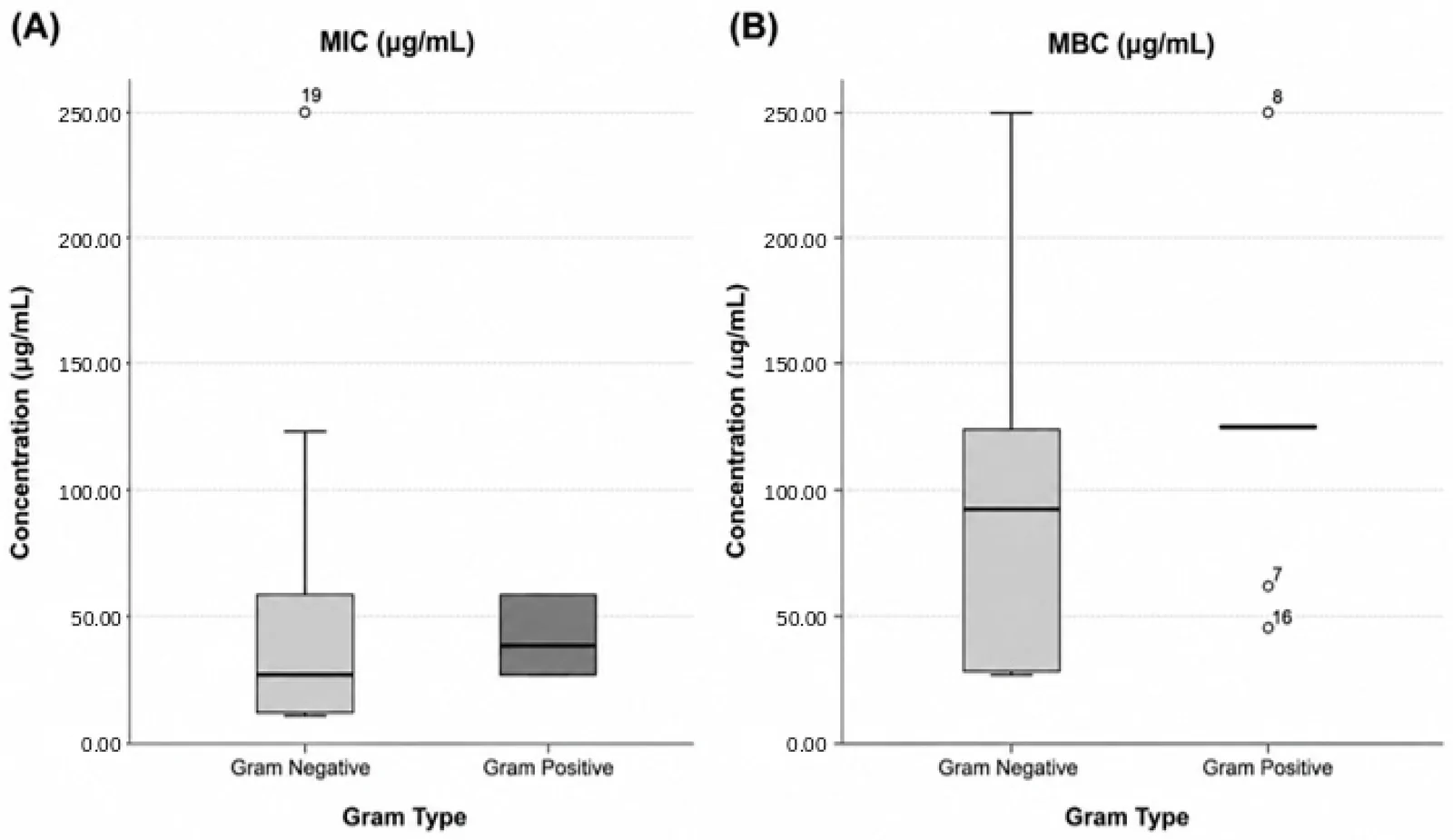
Distribution of MIC and MBC values of *L. orientalis* essential oil against Gram-positive and Gram-negative bacterial isolates. Panel (**A**) shows MIC values, whereas panel (**B**) shows MBC values. No statistically significant differences were observed between Gram-positive and Gram-negative groups for either MIC or MBC values (Mann–Whitney U test, *p* > 0.05). However, MBC values of Gram-positive bacteria were predominantly clustered around 125 µg/mL, indicating lower variability compared with Gram-negative isolates.

The distribution of MIC and MBC values among Gram-positive and Gram-negative bacterial isolates is presented in Figure 3. No statistically significant differences were observed between the two groups for either MIC or MBC values according to the Mann–Whitney U test (*p* > 0.05). Although MBC values of Gram-positive isolates appeared more tightly clustered around 125 µg/mL, indicating lower variability compared with Gram-negative isolates, this visual pattern was not supported by statistical significance. Likewise, no statistically significant association was observed between MIC values and MBC/MIC ratios, which were largely uniform across isolates, suggesting that bactericidal activity and biofilm inhibition may represent distinct antimicrobial properties under the tested conditions.

## 3. Discussion

The essential oil of *L. orientalis* has attracted considerable attention in traditional, complementary, and alternative medicine because of its diverse biological activities. Previous studies conducted in different regions have mainly focused on its antibacterial [7,20], wound-healing [21,22], antioxidant [8,22], and antiulcerogenic properties [9]. In Türkiye, *L. orientalis* has long been used in folk medicine, largely due to its reported antimicrobial, antioxidant, and antiulcerogenic activities [6,7]. However, most previous investigations evaluated the antibacterial activity of *L. orientalis* essential oil against only a limited number of bacterial species [7,8,23]. In contrast, the present study provides a more comprehensive evaluation of the antimicrobial potential of *L. orientalis* essential oil against a broad panel of clinically and veterinary relevant bacterial pathogens. A total of 24 bacterial isolates of Gram-positive and Gram-negative origin, including human, animal, and reference strains, were included in this study. Of these, 15 (62.5%) were Gram-negative and 9 (37.5%) were Gram-positive, while four isolates (16.7%) were identified as MDR (Table 7). The antibacterial activity of the essential oil was comprehensively evaluated using disk diffusion, well diffusion, MIC, MBC, and antibiotic essential oil synergy assays. In addition, its antibiofilm activity was investigated to assess its potential as a natural antimicrobial agent for controlling bacterial infections in both human and veterinary medicine.

Essential oils of aromatic and medicinal plants are complex mixtures of mono- and sesquiterpenes, phenylpropanoids, aromatic hydrocarbons, alcohols, aldehydes, ketones, and other oxygenated compounds, all of which may contribute to their biological properties and characteristic aroma [20]. In the current study, the essential oil of *L. orientalis* was characterized by a high proportion of styrene (68.6%), followed by (*E*)-ethyl cinnamate (6.8%), *α*-pinene (5.9%), *β*-pinene (2.9%), torreyol (1.6%), 4-ethylphenol (1.1%), and benzene propanol (0.9%), together with numerous mono and sesquiterpenes and aromatic derivatives present in lower concentrations (Table 1). Qualitative similarities were observed with previous reports on *L. orientalis*, which also identified aromatic derivatives and cinnamate-related compounds as important constituents [6]. Such compositional differences may be attributed to variations in geographical origin, harvesting season, plant genotype, physiological stage, or extraction conditions. Despite being the predominant constituent, styrene has only limited documented antibacterial activity, and its contribution to the overall antimicrobial effect of essential oils remains insufficiently understood. Therefore, the antibacterial activity observed in the present study is unlikely to be solely attributable to styrene. Instead, it is more plausibly explained by the combined effects of bioactive minor constituents, particularly *α*-pinene, (*E*)-ethyl cinnamate, 4-ethylphenol, and other oxygenated aromatic compounds, which have been previously associated with antimicrobial properties. Such additive or synergistic interactions among major and minor constituents are widely recognized as key determinants of the biological activity of complex essential oil mixtures and may account for the observed antibacterial effects. In addition, styrene is a highly volatile compound, and its stability and retention during agar diffusion assays (disk and well diffusion methods) may be limited under experimental conditions; therefore, potential losses due to volatilization or interactions with the solvent system may have affected its effective concentration in the agar medium, which should be considered a methodological limitation when interpreting the antimicrobial results.

The antibacterial activity of *L. orientalis* essential oil may primarily involve disruption of bacterial cell membrane integrity and increased membrane permeability, leading to leakage of intracellular components and loss of cellular viability [24]. In the present study, the complex mixture of aromatic compounds and terpenoids may have contributed to these effects through additive or synergistic interactions among multiple constituents. Such multifaceted interactions may also contribute to the observed bactericidal and antibiofilm effects, as essential oil constituents can act on multiple bacterial targets. These interactions may also enhance the activity of conventional antibiotics, as reported for essential oils and their constituents [25]. However, as specific mechanistic assays were not performed in the present study, these proposed mechanisms should be considered plausible rather than experimentally confirmed.

Essential oils derived from medicinal and aromatic plants have attracted considerable attention as natural antimicrobial agents due to their broad-spectrum activity against a wide range of pathogenic microorganisms. In this study, *L. orientalis* essential oil exhibited antibacterial activity against the tested bacterial strains, with an overall effectiveness of approximately 60.4% as determined by disk diffusion and agar well diffusion assays. The oil showed slightly greater activity against Gram-positive bacteria (77.8%) than Gram-negative bacteria (73.3%) (Figure 3). The strongest antibacterial effects were observed against *S. aureus* (14.3 ± 0.1 mm) in the disk diffusion assay and *B. melitensis* (16.4 ± 0.1 mm) in the agar well diffusion assay (Table 3 and Table 4). These findings are in agreement with those reported by Özcan Akyol and Doğanay [23], who demonstrated the antimicrobial efficacy of styrax liquidus, a resin derived from *L. orientalis*, against several nosocomial pathogens, including *P. aeruginosa*, *K. pneumoniae*, *S. haemolyticus*, *A. baumannii*, and *E. faecalis*. The relatively higher susceptibility of Gram-positive bacteria may be explained by their cell wall structure, which consists mainly of a thick peptidoglycan layer that is more accessible to hydrophobic essential oil constituents. In contrast, the outer membrane rich in lipopolysaccharides found in Gram-negative bacteria can limit the penetration of antimicrobial compounds [26]. Overall, the present results support the broad-spectrum antimicrobial potential of *L. orientalis* essential oil and are consistent with previous reports on its antibacterial properties. Different solvents were used according to the requirements of each assay, with DMSO used for MIC determinations and ethanol for the disk diffusion and agar well diffusion assays. To exclude any solvent-related effects, corresponding solvent controls (1% DMSO or ethanol) were included in all experimental setups. As no inhibitory activity was observed in the solvent controls, the antibacterial effects reported in this study can be attributed to the *L. orientalis* essential oil rather than to the solvents used.

The antibacterial efficacy of *L. orientalis* essential oil was evaluated against a panel of bacterial pathogens. Our findings demonstrated that *L. orientalis* essential oil exhibited antibacterial activity against the majority of the bacterial species tested, although the degree of susceptibility varied among the isolates. The lowest MIC (15.62 µg/mL) and MBC (31.25 µg/mL) values were observed for *E. coli* and *B. melitensis*, whereas the highest MIC (250.00 µg/mL) and MBC (250.00 µg/mL) values were recorded for human-derived *S. aureus* and human-derived *P. aeruginosa* (Table 2, Figure 2). These findings indicate species-dependent differences in susceptibility to *L. orientalis* essential oil and are consistent with previous reports describing variable antibacterial responses to plant-derived essential oils [27]. Although Gram-positive isolates tended to exhibit numerically lower MIC and MBC values than Gram-negative isolates (Figure 3), these differences were not statistically significant, indicating comparable antibacterial activity between the two bacterial groups. The numerically lower susceptibility observed in some Gram-negative isolates may be explained by the presence of an outer membrane enriched with lipopolysaccharides, which acts as an additional permeability barrier and restricts the diffusion of hydrophobic essential oil constituents. Conversely, the absence of this outer membrane in Gram-positive bacteria may facilitate interactions between essential oil components and the cytoplasmic membrane, potentially contributing to the observed numerical trend. A strong positive correlation was observed between MIC and MBC values (Spearman’s ρ = 0.883, *p* < 0.001), indicating that isolates requiring higher concentrations for growth inhibition also required proportionally higher concentrations for bactericidal activity. Furthermore, the mean MBC/MIC ratio was 2.29 ± 0.72, and all ratios were ≤4, confirming the bactericidal activity of *L. orientalis* essential oil against all susceptible isolates (Figure 2). Collectively, these findings demonstrate the broad-spectrum antibacterial potential of *L. orientalis* essential oil and support its potential as a promising natural antimicrobial agent against both Gram-positive and Gram-negative bacteria.

Synergistic interactions between essential oils (EOs) and conventional antibiotics have attracted considerable attention as a promising strategy to enhance antimicrobial efficacy and overcome resistance mechanisms in pathogenic microorganisms [28]. Previous studies have reported synergistic, additive, indifferent, or antagonistic interactions depending on the bacterial species, antibiotic class, and EO composition [4,20,29]. In the present study, *L. orientalis* essential oil exhibited variable interactions with different antibiotics against both Gram-positive and Gram-negative bacteria, including MDR strains. Synergistic effects were observed in several combinations, particularly against MDR *S. aureus* (amoxicillin), *L. monocytogenes* (amoxicillin and erythromycin), *S.* Typhimurium (enrofloxacin), and *B. melitensis* (tetracycline and gentamicin), whereas MDR *E. coli* exhibited indifferent (gentamicin) or antagonistic (enrofloxacin) interactions (Table 5). The antagonistic effect observed with enrofloxacin against MDR *E. coli* may be attributed to interference between essential oil constituents and antibiotic activity, potentially affecting antibiotic uptake or intracellular availability. Overall, these findings demonstrate that the interaction between *L. orientalis* essential oil and conventional antibiotics is strain- and antibiotic-dependent, highlighting the importance of evaluating each EO-antibiotic combination individually before considering therapeutic applications.

Brucellosis is a widespread zoonotic disease caused by various *Brucella* species. In humans, *B. melitensis* is the most pathogenic species, followed by *B. suis*, while *B. abortus* is generally associated with milder forms of the disease. It mainly affects livestock and wildlife and poses significant public health threats, especially in regions with suboptimal hygiene, food safety, and veterinary care standards. Human infections occur through consumption of contaminated animal products or interaction with infected animals [30,31]. Antibiotics play a major role in the management of brucellosis in humans. However, as with many bacterial pathogens, resistance to antibiotics used against *Brucella* infections has been reported [29,32]. It has been stated [31] that, although a single antibiotic may be effective, combination therapy is preferred to reduce the risk of resistance development and disease recurrence. In this study, considering that the volatile constituents of the essential oil of *L. orientalis* showed significant in vitro activity against *B. melitensis* in both tests (Table 3 and Table 4), and that synergistic effects between herbal essential oils and antibiotics have been reported in the context of brucellosis management [33], we hypothesized that combining *L. orientalis* essential oil with antibiotics may have potential as an adjunct approach against *B. melitensis* infections. However, this suggestion is based on in vitro findings against a single *B. melitensis* strain, and further in vivo and clinical studies are required to evaluate its therapeutic potential.

The exopolysaccharide (EPS) matrix of bacterial biofilms and the increased antimicrobial tolerance of biofilm-associated cells considerably reduce the efficacy of conventional therapies. Consequently, the development of agents capable of preventing biofilm formation or disrupting established biofilms has become an important strategy for controlling healthcare-associated and device-related infections [34]. Information regarding the antibiofilm activity of *L. orientalis* essential oil remains limited. Mutlu et al. [35] demonstrated that this essential oil inhibited biofilm formation by *P. aeruginosa* and *S. aureus*, particularly at relatively low concentrations. Consistent with these findings, *L. orientalis* essential oil exhibited antibiofilm activity against all tested biofilm-forming bacteria in the present study, although the extent of inhibition varied markedly among species and strains (Table 6). This variability suggests that the antibiofilm efficacy of the essential oil is strain-dependent and may reflect differences in biofilm architecture, EPS composition, or intrinsic bacterial characteristics. No significant correlations were observed between antibiofilm activity and either MIC or MBC values, indicating that the mechanisms underlying biofilm inhibition may not directly parallel those responsible for planktonic antibacterial activity.

Disk diffusion and agar well diffusion assays are among the most widely used screening methods for evaluating the antibacterial activity of plant-derived essential oils [8,20,23]. In the present study, the two methods showed broadly similar patterns of antibacterial activity, although the inhibition zone diameters obtained by the two assays should not be considered directly quantitatively equivalent because the diffusion conditions differed between the methods. In particular, the two assays used different diffusion sources and volumes of essential oil (10 µL per 6-mm paper disc in the disk diffusion assay and 50 µL per 7-mm agar well in the agar well diffusion assay), which may influence the extent of diffusion and the resulting inhibition zones. Therefore, the comparison between the two methods in the present study was intended to assess overall patterns of antibacterial activity and practical applicability rather than to establish quantitative equivalence between the assays. The choice of method for preliminary screening may consequently be influenced by practical considerations, including laboratory resources, sample volume, and experimental design. Although MDR status did not significantly influence susceptibility in the disk diffusion assay, MDR isolates showed reduced susceptibility in the agar well diffusion assay, suggesting that methodological factors may influence the detection of antibacterial activity against resistant strains.

## 4. Materials and Methods

### 4.1. Sample Preparation

*L. orientalis* (storax) balsam used in this study was obtained from a local producer in Muğla, Türkiye. The essential oil was extracted from the balsam by hydrodistillation for 3 h using a Clevenger-type apparatus. The resulting essential oil was subsequently subjected to gas chromatography-mass spectrometry (GC-MS) and gas chromatography-flame ionization detection (GC-FID) analyses and was also used for the antibacterial assays. GC-MS and GC-FID analyses were carried out at the Plant, Drug and Scientific Research Application and Research Center (AUBİBAM), Anadolu University, Eskişehir, Türkiye, using an Agilent 7890B GC System (G3440B, Agilent Technologies, Santa Clara, CA, USA). Prior to analysis, the essential oil was diluted to 10% (*w*/*w*) in hexane, and a 1 μL aliquot was injected into the GC system using a split ratio of 40:1.

### 4.2. GC-MS and GC-FID Analysis of L. orientalis Essential Oil

The relative composition of volatile compounds was determined by GC-FID using an Agilent 7890B GC system equipped with an HP-Innowax capillary column (60 m × 0.25 mm i.d., 0.25 µm film thickness). The injector and detector temperatures were maintained at 250 °C. The oven temperature was programmed from 60 °C (held for 10 min) to 220 °C at 4 °C/min (held for 10 min), followed by an increase to 240 °C at 1 °C/min. Helium was used as the carrier gas at a constant flow rate of 0.7 mL/min. Relative percentages of the components were calculated from FID peak areas without applying correction factors.

Volatile compounds were identified by GC-MS using an Agilent 7890B GC coupled to a 5977B MSD, operated in EI mode at 70 eV under the same chromatographic conditions. The ion source temperature was maintained at 230 °C, and mass spectra were recorded over the range of 35–450 *m*/*z*. Compound identification was performed based on mass spectral matching with the Wiley 9 and NIST 11 libraries, followed by comparison of the calculated linear retention indices (LRIc) with literature retention index values (LRIr) compiled from original published studies [9,10,11,12,13,14,15,16,17,18,19].

### 4.3. Bacterial Strains

A total of 24 bacterial strains stored at −80 °C were used in this study. The strains were selected based on their clinical significance and antimicrobial resistance profiles (Table 7) and were obtained from the Department of Microbiology, Faculty of Veterinary Medicine, Balıkesir University. Prior to the experiments, all bacterial strains were cultured in appropriate liquid media: all strains except *B. melitensis* were grown aerobically in Mueller-Hinton broth (MHB, 211443, BD, Sparks, MD, USA) at 37 °C for 18–24 h, whereas *B. melitensis* strains were cultured in tryptic soy broth (TSB, 1.05459, Merck, Darmstadt, Germany) at 37 °C for 24–36 h. For the determination of antimicrobial susceptibility profiles, the Kirby–Bauer disk diffusion method [36] was performed in accordance with Clinical and Laboratory Standards Institute guidelines [37]. The turbidity of all cultures was adjusted to 0.5 McFarland standards using sterile saline. Subsequently, 100 µL of each bacterial suspension was spread onto Mueller-Hinton agar (MHA; 1.103872, Merck, Darmstadt, Germany) plates; plates supplemented with 5% defibrinated sheep blood were used for *B. melitensis*. After drying at room temperature for 30 min, the following selected antibiotic disks were used: chloramphenicol (C, 10 µg, CT0013B, Oxoid, Basingstoke, UK), norfloxacin (NOR, 10 µg, CT0434B, Oxoid), streptomycin (S10; CT0047B, Oxoid), ceftiofur (EFT, 30 µg, CT1751B, Oxoid), tetracycline (TE, 30 µg, CT0054B, Oxoid), ampicillin/sulbactam (SAM, 20 µg, CT0520B, Oxoid), ciprofloxacin (CIP, 10 µg, CT1615B, Oxoid), erythromycin (E, 15 µg, CT0020B, Oxoid), and cefoperazone (CFP, 75 µg, CT02449B, Oxoid). The plates were incubated at 37 °C for 18–24 h, whereas *B. melitensis* plates were incubated for 24–36 h.

**Table 7:** Bacterial strains, their sources and antibiotic resistance profiles *.

	Bacteria	Strain Origin	Antimicrobial Resistance Profile
1	*Escherichia coli*	ATCC 25922	C(S), NOR(S), S(R), EFT(S), TE(I), SAM(S), CIP(S), E(I), CFP(S)
2	MDR *Escherichia coli*	Animal-derived (culture collection)	C(R), NOR(S), S(R), EFT(R), TE(R), SAM(R), CIP(S), E(S), CFP(S)
3	*Escherichia coli*	Human-derived (culture collection)	C(S), NOR(S), S(I), EFT(I), TE(S), SAM(S), CIP(S), E(S), CFP(S)
4	*Escherichia coli*	Animal-derived (culture collection)	C(S), NOR(S), S(R), EFT(S), TE(S), SAM(S), CIP(S), E(R), CFP(S)
5	*Staphylococcus aureus* subsp. *aureus*	ATCC 6538	C(R), NOR(S), S(R), EFT(S), TE(S), SAM(S), CIP(S), E(S), CFP(S)
6	MRSA	ATCC 43300	C(S), NOR(S), S(R), EFT(S), TE(R), SAM(R), CIP(S), E(R), CFP(S)
7	MDR *S. aureus*	Animal-derived (culture collection)	C(R), NOR(R), S(R), EFT(S), TE(R), SAM(S), CIP(S), E(I), CFP(S)
8	*Staphylococcus aureus*	Human-derived (culture collection)	C(S), NOR(S), S(S), EFT(S), TE(R), SAM(S), CIP(S), E(R), CFP(I)
9	*Staphylococcus aureus*	Animal-derived (culture collection)	C(R), NOR(S), S(I), EFT(S), TE(S), SAM(S), CIP(S), E(S), CFP(S)
10	*Enterococcus faecalis*	ATCC 29212	C(S), NOR(S), S(I), EFT(S), TE(S), SAM(I), CIP(S), E(R), CFP(S)
11	*Enterococcus faecium*	ATCC 6057	C(S), NOR(R), S(R), EFT(S), TE(I), SAM(S), CIP(S), E(S), CFP(S)
12	*Klebsiella pneumoniae*	Human-derived (culture collection)	C(S), NOR(S), S(I), EFT(S), TE(S), SAM(S), CIP(S), E(S), CFP(R)
13	*Klebsiella pneumoniae*	Animal-derived (culture collection)	C(S), NOR(S), S(I), EFT(R), TE(S), SAM(S), CIP(S), E(R), CFP(S)
14	*Acinetobacter baumannii*	Human-derived (culture collection)	C(S), NOR(S), S(I), EFT(S), TE(S), SAM(R), CIP(S), E(S), CFP(R)
15	*Acinetobacter baumannii*	Human-derived (culture collection)	C(I), NOR(R), S(I), EFT(S), TE(R), SAM(S), CIP(S), E(R), CFP(S)
16	*Listeria monocytogenes*	ATCC 7644	C(I), NOR(S), S(S), EFT(S), TE(S), SAM(R), CIP(S), E(S), CFP(S)
17	*Salmonella enterica* subsp. *enterica* serovar Typhimurium	NCTC 12416	C(R), NOR(I), S(R), EFT(S), TE(S), SAM(S), CIP(S), E(R), CFP(S)
18	*Pseudomonas aeruginosa*	Human-derived (culture collection)	C(S), NOR(S), S(R), EFT(S), TE(S), SAM(R), CIP(S), E(I), CFP(S)
19	MDR *Pseudomonas aeruginosa*	Animal-derived (culture collection)	C(I), NOR(S), S(R), EFT(S), TE(R), SAM(R), CIP(S), E(R), CFP(S)
20	*Mannheimia haemolytica*	Human-derived (culture collection)	C (R), NOR(S), S(S), EFT(S), TE(I), SAM(S), CIP(S), E(S), CFP(S)
21	*Mannheimia haemolytica*	Animal-derived (culture collection)	C(S), NOR(S), S(S), EFT(S), TE(R), SAM(S), CIP(S), E(S), CFP(S)
22	*Brucella melitensis* strain 16M (biotype 1)	ATCC 23456	C(S), NOR(S), S(R), EFT(I), TE(S), SAM(R), CIP(S), E(S), CFP(S)
23	*Brucella melitensis*	Animal-derived (culture collection)	C(R), NOR(S), S(R), EFT(S), TE(S), SAM(S), CIP(S), E(S), CFP(S)
24	*Corynebacterium pseudotuberculosis*	Animal-derived (culture collection)	C(S), NOR(S), S(R), EFT(S), TE(S), SAM(R), CIP(S), E(S), CFP(I)

* All human- and animal-derived bacterial strains were previously isolated and obtained from the institutional culture collection. The isolates were anonymized, and this study did not involve new clinical specimens, human participants, or animals. Abbreviations: MDR: multidrug-resistant; isolates were classified according to their individual resistance profiles, and MDR was defined as resistance to ≥3 different classes of antibacterial agents. MRSA: Methicillin-resistant *Staphylococcus aureus*; C: chloramphenicol; NOR: norfloxacin; S: streptomycin; EFT: ceftiofur; TE: tetracycline; SAM: ampicillin/sulbactam; CIP: ciprofloxacin; E: erythromycin; CFP: cefoperazone; S: Susceptible; I: Intermediate; R: Resistant.

### 4.4. Evaluation of Antibacterial Activity

#### 4.4.1. Determination of Minimum Inhibitory Concentration (MIC) and Minimum Bactericidal Concentration (MBC) of *L. orientalis* Essential Oil

The minimal inhibitory concentration (MIC) of *L. orientalis* essential oil was determined using a two-fold serial microdilution method according to the Clinical and Laboratory Standards Institute guideline [38], with slight modifications. *L. orientalis* essential oil was serially diluted in dimethyl sulfoxide (DMSO; A994.2, Karlsruhe, Germany) to obtain concentrations of 250, 125, 62.5, 31.25, 15.62, 7.81, 3.9, and 1.95 µg/mL. Each dilution was prepared in MHB containing 1% DMSO. Then, 100 µL of each dilution was mixed with 100 µL of bacterial suspension (10^8^ CFU/mL) in 96-well microplates (Costar 3599, Coning, NY, USA). A 1% DMSO solution was used as the negative control. The microplates were incubated at 37 °C for 24 h, and bacterial growth was assessed by measuring optical density at 600 nm using a microplate reader (Spectrostar Nano, BMG LabTech, Ortenberg, Germany). MIC was defined as the lowest concentration that resulted in inhibition of bacterial growth compared with the untreated growth control.

Percentage inhibition was calculated using the following equation:

$$\%Inhibition=\left(1-\frac{ODsample-ODblank}{ODgrowth-ODblank}\right)\times 100$$

For MBC determination, 30 µL aliquots from all wells showing no visible growth in the MIC assay were subcultured onto MHA plates and incubated at 37 °C for 24 h. The MBC was defined as the lowest concentration of the essential oil that resulted in no visible bacterial growth on agar after subculture. A bacterial suspension of 10^8^ CFU/mL was used as the initial inoculum, and the same inoculum was applied consistently across all assays. Gentamicin sulfate (1405-41-0, Sigma-Aldrich, Seelze, Germany) and streptomycin sulfate (Cat no. 2311004, Menarini Health and Pharmaceutical Industry Trade Inc., Istanbul, Turkey) were used as standard antibacterial agents, while 1% DMSO served as the negative control.

#### 4.4.2. Disk Diffusion Assay

The disk diffusion assay was performed using the Kirby–Bauer method [36] according to Clinical and Laboratory Standards Institute guidelines [37]. *L. orientalis* essential oil was dissolved in absolute ethanol (≥99.8%, 32221, Sigma-Aldrich, Steinheim, Germany) to prepare concentrations of 250–1.95 µg/mL. Sterile 6 mm filter paper disks (CT0998B, Oxoid, Basingstoke, UK) were impregnated with 20 µL of each *L. orientalis* essential oil solution. Bacterial strains, except *B. melitensis*, were cultured aerobically in MHB at 37 °C for 18–24 h. *B. melitensis* strains were cultured in TSB at 37 °C for 24–36 h. The turbidity of all cultures was adjusted to 0.5 McFarland standards using sterile saline solution. Subsequently, 100 µL of bacterial suspension was spread onto MHA plates; agar supplemented with 5% defibrinated sheep blood was used for *B. melitensis*. After drying at room temperature for 30 min, the impregnated disks were placed on the agar surface and plates were incubated at 37 °C for 18–24 h. Disks impregnated with absolute ethanol (Sigma-Aldrich) served as negative controls [39], and gentamicin (CN, 10 μg, Oxoid, Basingstoke, UK) and streptomycin (S, 10 μg, Oxoid, Basingstoke, UK) disks were used as positive controls. All experiments were performed in duplicate, and inhibition zone diameters, including the disk diameter, were measured in millimeters. Based on in-house standardization experiments, a 7 mm inhibition zone was obtained with disks impregnated with *L. orientalis* essential oil at a concentration of 31.25 µg/mL. Therefore, inhibition zones greater than 7 mm were considered indicative of antibacterial activity.

#### 4.4.3. Agar Well Diffusion Assay

The agar well diffusion assay was performed according to a modified agar well diffusion method previously described [40]. A total of 30 mL of MHA was poured into 100 mm Petri dishes, and 6 mm-diameter wells with a depth of 5 mm were created after the agar had solidified. Bacterial suspensions (100 µL), adjusted to the 0.5 McFarland standard, were evenly spread over the agar surface using a sterile swab. *L. orientalis* essential oil was dissolved in absolute ethanol (Sigma-Aldrich) to prepare concentrations of 250–1.95 µg/mL, and 50 µL of each solution was added to the wells. Ethanol was used as a negative control [41], and gentamicin sulfate and streptomycin sulfate were used as positive controls. The plates were incubated at 37 °C for 24 h in a humidified chamber. Following incubation, inhibition zone diameters, including the well diameter, were measured in millimeters. Based on in-house standardization experiments, a 6 mm inhibition zone was obtained with *L. orientalis* essential oil at a concentration of 31.25 µg/mL. Therefore, inhibition zones greater than 6 mm were considered indicative of antibacterial activity. All experiments were performed in duplicate.

### 4.5. Synergistic Effect of L. orientalis Essential Oil and Antibiotics

The synergistic interactions between *L. orientalis* essential oil and selected antibiotics were evaluated using the broth microdilution checkerboard assay with slight modifications, according to the method described by Fratini et al. [42]. Erythromycin A dehydrate (E, CAS No. 59319-72-1, Sigma-Aldrich, Seelze, Germany) and amoxicillin trihydrate (AM, CAS No. 26787-78-0, Sigma-Aldrich) were tested against MDR *S. aureus* and *L. monocytogenes* (ATCC 7644). Enrofloxacin (ENR, CAS No. 93106-60-6, Sigma-Aldrich) and gentamicin sulfate (CN, CAS No. 1405-41-0, Sigma-Aldrich) were tested against MDR *E. coli* and *S*. Typhimurium NCTC 12416, whereas tetracycline hydrochloride (TE, CAS No. 64-75-5, Sigma-Aldrich) and gentamicin sulfate were evaluated against an animal-derived strain of *B. melitensis*. The selection of antibiotic combinations was based on the clinical relevance of these agents in the treatment of infections caused by the corresponding pathogens in both humans and animals. Accordingly, tetracycline is commonly used in the management of *Brucella* infections, while enrofloxacin and gentamicin are frequently applied against *E. coli* and *Salmonella* infections. In addition, amoxicillin and erythromycin are widely used for infections caused by *S. aureus* and *L. monocytogenes*, respectively.

Two-fold serial dilutions of antibiotics (1024–0.125 µg/mL) and *L. orientalis* essential oil (250–1.95 µg/mL) were prepared. Briefly, 100 µL of sterile MHB was dispensed into each well of a sterile 96-well microtiter plate. Antibiotics were added to the first column and serially diluted horizontally across the plate, while *L. orientalis* essential oil was added to the first row and serially diluted vertically, generating different combinations of antibiotic and essential oil concentrations. Subsequently, 100 µL of the standardized bacterial suspension was added to each well. The microplates were incubated at 35 °C for 18–24 h. Following incubation, 30 µL aliquots from each well were streaked onto MHA plates and incubated at 35 °C for an additional 18 h. All experiments were performed independently in triplicate to ensure the reproducibility and reliability of the results.

### 4.6. Fractional Inhibitory Concentration Index (FICI) Analysis

The FICI of *L. orientalis* essential oil combined with the tested antibiotics was calculated to evaluate their interactions against selected bacterial strains. The FICI was calculated using the following formula:

$$FICI=\frac{MIC_{LOEO+AB}}{MIC_{LOEO}}+\frac{MIC_{AB+LOEO}}{MIC_{AB}}$$

LOEO: *L. orientalis* essential oil, AB: Antibiotic.

The FICI values were interpreted as a synergistic effect when FICI ≤ 0.5; an additive effect when 0.5 < FICI < 1; indifferent when 1 < FICI < 4; and an antagonistic effect when FICI > 4.

### 4.7. Evaluation of Antibiofilm Activity

Antibiofilm activity of *L. orientalis* essential oil was evaluated using a modified microtiter plate method based on the protocol described by O’Toole [43]. Briefly, bacterial cultures were grown in TSB at 37 °C for 24 h, and the inoculum density was adjusted to the 0.5 McFarland standard. Based on the MIC values determined for each bacterial strain, a strain-specific sub-MIC of *L. orientalis* essential oil was selected for the antibiofilm assay. This approach was used to ensure that the tested concentration was below the inhibitory concentration for each strain. For the assay, 100 µL of TSB containing the strain-specific sub-MIC of *L. orientalis* essential oil was added to each well of a sterile, flat-bottom 96-well microtiter plate, followed by 100 µL of the bacterial suspension. The essential oil was prepared in MHB containing <1% DMSO. The plates were incubated at 37 °C for 24 h to allow biofilm formation. After incubation, the culture medium was carefully removed, and the wells were washed three times with sterile distilled water and allowed to air-dry. Subsequently, 100 µL of 0.1% crystal violet solution (1.15940, Merck, Darmstadt, Germany) was added to each well, and the plates were incubated at room temperature for 15 min. Excess stain was removed by washing the wells three times with sterile distilled water, and the plates were allowed to dry completely. The bound crystal violet was then solubilized by adding 200 µL of an ethanol/acetone solution (80:20, *v*/*v*) to each well and incubating for 10 min at room temperature. Biofilm biomass was quantified by measuring the absorbance at 570 nm using a microplate reader. All experiments were performed in duplicate. Sterile saline was used as the negative control, while *Bacillus subtilis* ATCC 6633 was used as the biofilm-positive control.

Biofilm inhibition (%) was calculated using the following equation:

$$\%Biofilm\;Inhibition=\;\left(1-\;\frac{ODsample}{ODcontrol}\right)\;\times 10$$

### 4.8. Statistical Analysis

Statistical analyses were performed using IBM SPSS Statistics for Windows, Version 26.0 (IBM Corp., Armonk, NY, USA). Descriptive data are presented as mean ± standard deviation (SD). Data normality was assessed using the Shapiro–Wilk test. As MIC and MBC values were not normally distributed and the sample size was limited, non-parametric tests were used throughout the study. Comparisons between two independent groups were performed using the Mann–Whitney U test, whereas comparisons among three groups were conducted using the Kruskal–Wallis test. Effect sizes were calculated as r for the Mann–Whitney U test and eta-squared (η^2^) for the Kruskal–Wallis test. Agreement between the disk diffusion and agar well diffusion methods was evaluated using McNemar’s exact test, while associations between categorical variables were assessed using Fisher’s exact test. Correlations among MIC, MBC, biofilm inhibition, and MBC/MIC ratio values were evaluated using Spearman’s rank correlation coefficient (ρ). A *p*-value < 0.05 was considered statistically significant. The antibacterial effect of the essential oil was classified as bactericidal or bacteriostatic based on the MBC/MIC ratio. Ratios ≤4 were considered bactericidal, whereas ratios >4 were considered bacteriostatic, according to previously established criteria [44,45].

**Figure 4:**
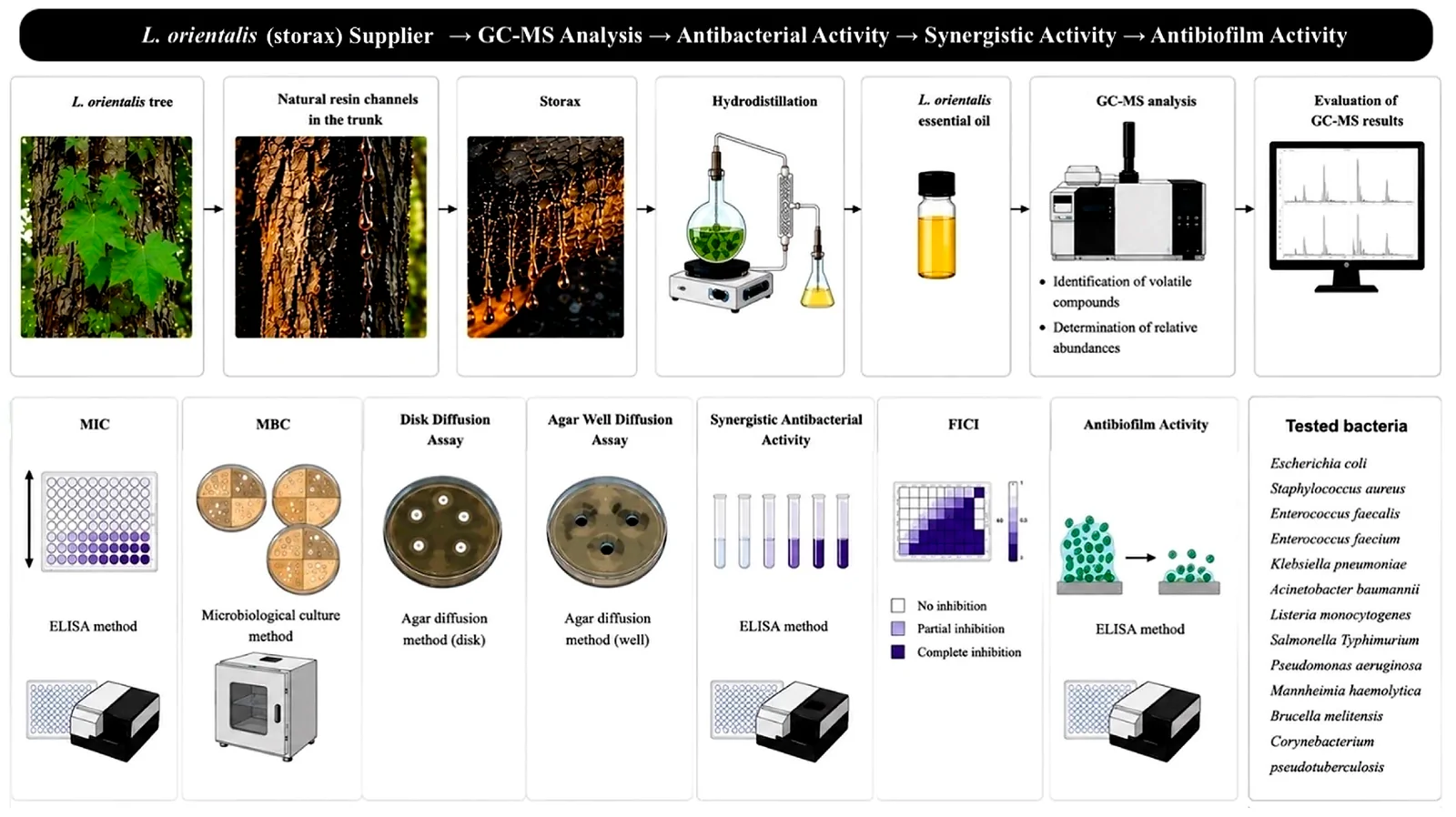
Schematic overview of the production of *L. orientalis* (storax) balsam by the supplier, followed by GC-MS analysis and evaluation of its antibacterial, synergistic, and antibiofilm activities.

A schematic overview of the study workflow, including the supplier extraction of *L. orientalis* essential oil, GC-MS analysis, and antibacterial, synergistic, and antibiofilm activities, is presented in Figure 4.

## 5. Conclusions

The findings of this study demonstrate that *L. orientalis* essential oil possesses broad-spectrum antibacterial and antibiofilm activities against a range of clinically significant pathogens, including *E. coli*, *S. aureus*, *E. faecium*, *K. pneumoniae*, *M. haemolytica*, and particularly *B. melitensis*. The essential oil exhibited in vitro antibacterial activity at low concentrations (15.62–31.25 µg/mL). However, its activity against MDR strains was variable depending on the organism and assay conditions. While no inhibition was observed against MDR *P. aeruginosa* in the agar well diffusion assay, MDR *E. coli* showed variable responses, and only limited inhibition was observed against MDR *S. aureus* in the disk diffusion assay (6.8 mm, below the activity threshold). Furthermore, synergistic effects with conventional antibiotics suggest potential as an adjunct approach rather than direct therapeutic efficacy. Overall, these findings indicate that *L. orientalis* essential oil may serve as a promising candidate for further investigation in the development of alternative strategies against antibiotic-resistant infections in both humans and animals. Nevertheless, further studies, including toxicity assessments, mechanism of action investigations, and in vivo evaluations, are required to confirm its safety, efficacy, and therapeutic potential before any clinical application.

## Figures and Tables

**Table 1:** Chemical composition of *L. orientalis* essential oil identified by GC-MS analysis.

No	Compounds	LRIc	LRIr	Reference	Relative Percentage (%)
1	*α*-Pinene	1030	1032 ^a,b,c^ 1008–1039 ^b^	[9,10,11]	5.9
2	Camphene	1080	1076 ^a,d,e^ 1043–1086 ^b^	[9,10,12,13]	0.4
3	*β*-Pinene	1118	1118 ^a,e,f^ 1085–1130 ^b^	[9,10,13,14]	2.9
4	Sabinene	1129	1132 ^e,g^ 1098–1140 ^b^	[10,13,15]	0.2
5	Myrcene	1168	1174 ^e,h^ 1140–1175 ^b^	[10,13,16]	0.7
6	*α*-Phellandrene	1170	1176 ^e,h^ 1148–1186 ^b^	[10,13,16]	tr
7	*α*-Terpinene	1185	1154–1195 ^b^	[10]	tr
8	Limonene	1205	1203 ^a,e,h^ 1178–1219 ^b^	[9,10,13,16]	0.5
9	*β*-Phellandrene	1214	1218 ^e,h^ 1188–1233 ^b^	[10,13,16]	0.2
10	1,8-Cineole	1217	1213 ^a^ 1186–1231 ^b^	[9,10]	0.2
11	(*Z*)-*β*-Ocimene	1238	1246 ^h^ 1211–125 ^b^	[10,16]	tr
12	γ-Terpinene	1251	1255 ^a^ 1222–1266 ^b^	[9,10]	tr
13	(*E*)-*β*-Ocimene	1256	1266 ^e,h^ 1232–1267 ^b^	[10,13,16]	0.1
14	Styrene	1265	1272 ^a,c^ 1240–1290 ^b^	[9,10,11]	68.6
15	Terpinolene	1284	1290 ^e^ 1261–1300 ^b^	[10,13]	tr
16	Tridecane	1300	1300 ^h^	[16]	tr
17	(*E*)-4,8-Dimethyl-1,3,7-nonatriene	1308	1309 ^k^	[17]	tr
18	Nonanal	1398	1370–1414 ^b^	[10]	tr
19	Propenyl benzene	1421	-	-	tr
20	*α*-Cubebene	1463	1466 ^c^ 1438–1480 ^b^	[10,11]	0.2
21	*trans*-Sabinene hydrate	1469	1474 ^f^	[14]	tr
22	Cyclosativene	1492	1445–1549 ^b^	[10]	tr
23	*α*-Copaene	1498	1497 ^a,c^ 1462–1522 ^b^	[9,10,11]	tr
24	*β*-Cubebene	1544	1518–1560 ^b^	[10]	0.5
25	Benzaldehyde	1547	1541 ^a^ 1481–1555 ^b^	[9,10]	0.4
26	*cis*-Sabinene hydrate	1553	1556 ^f^	[14]	tr
27	Linalyl acetate	1556	1532–1570 ^b^	[10]	tr
28	*cis*-Myrtanal	1565	1560 ^d^	[12]	0.1
29	*β*-Ylangene	1581	1547–1589 ^b^	[10]	tr
30	*trans*-Myrtanal	1584	1570 ^d^	[12]	0.1
31	Bornyl acetate	1587	1590–1591 ^a,c,e^	[9,11,13]	0.1
32	*β*-Elemene	1593	1600 ^c,e^ 1565–1608 ^b^	[10,11,13]	tr
33	*β*-Copaene	1600	1602 ^c^ 1550–1603 ^b^	[10,11]	0.5
34	*β*-Caryophyllene	1606	1569–1632 ^b^	[10]	0.1
35	Terpinen-4-ol	1609	1611 ^a,h^ 1564–1630 ^b^	[9,10,16]	0.1
36	Alloaromadendrene	1656	166 ^c^ 1624–1668 ^b^	[10,11]	0.1
37	(*Z*)-*β*-Farnesene	1663	1627–1668 ^b,e^	[10,13]	tr
38	Acetophenone	1673	1671 ^a^ 1607–1699 ^b^	[9,10]	0.7
39	Methyl chavicol	1683	1652–1690 ^b^	[10]	tr
40	*γ*-Muurolene	1696	1704 ^c^ 1655–1714 ^b^	[10,11]	0.3
41	*α*-Terpineol	1703	1706 ^a,h^ 1659–1724 ^b^	[9,10,16]	0.1
42	Borneol	1710	1719 ^a^ 1653–1728 ^b^	[9,10]	tr
43	*cis*-4,10-epoxy-Amorphane	1713	-	-	0.2
44	Germacrene D	1727	1676–1726 ^b,e^	[10,13]	0.6
45	Bicyclosesquiphellandrene	1733	1722 ^c^	[11]	tr
46	*α*-Muurolene	1735	1740 ^a,c^ 1686–1753 ^b^	[9,10,11]	0.3
47	Verbenone	1738	1696–1735 ^b^	[10]	tr
48	*δ*-Cadinene	1766	1773 ^a,e^ 1722–1774 ^b^	[9,10,13]	0.2
49	*γ*-Cadinene	1773	1776 ^c^ 1735–1782 ^b^	[10,11]	tr
50	*β*-Sesquiphellandrene	1780	1748–1783 ^b^	[10]	tr
51	3-Chromene	1787	-	-	tr
52	Cubenene	1798	1764–1810 ^b^	[12]	tr
53	Benzene propanal	1805	1800 ^a^	[9]	0.7
54	1-Phenyl-1,2-propanedione	1835	1817 ^m^	[17]	tr
55	*cis*-Calamenene	1850	1849 ^c^	[13]	tr
56	(*E*)-Geranylacetone	1865	1820–1873 ^b^	[10]	tr
57	2,5-Dimethoxy-p-cymene	1872	1826–1878 ^b^	[10]	tr
58	Ethyl 3-phenylpropionate	1898	1900 ^c^	[11]	0.1
59	*epi*-Cubebol	1902	1904 ^c^ 1854–1928 ^b^	[10,11]	0.1
60	3-Phenylpropyl acetate (Hydrocinnamyl acetate)	1921	1912 ^c^, 1872 ^g^	[11,15]	tr
61	Cubebol	1957	1933 ^c^ 1884–1964 ^b^	[10,11]	0.2
62	Vinyl phenyl carbinol	1988	-	-	0.4
63	(*Z*)-Ethyl cinnamate	2011	2012 ^m^	[18]	tr
64	4,10(14)-Muuroladien-8β-ol	2013	-	-	tr
65	Methyl eugenol	2024	2030 ^h^ 1961–2033 ^b^	[10,16]	tr
66	(*E*)-Nerolidol	2038	1995–2055 ^b^	[10]	tr
67	Salvial-4(14)-en-1-one	2040	2037 ^c^ 2016–2043 ^b^	[10,11]	tr
68	4-Ethyl guaiacol	2045	2049 ^a^ 1986–2065 ^b^	[9,10]	tr
69	Benzene propanol	2061	2065 ^a^	[9]	0.9
70	(*E*)-Cinnamaldehyde	2081	2068 ^a^, 2069 ^c^ 2015–2044 ^b^	[9,10,11]	0.5
71	Elemol	2094	2043–2103 ^b^	[10]	0.1
72	Methyl cinnamate	2106	2096 ^a,c^ 2046–2105 ^b^	[9,10,11]	0.4
73	Spathulenol	2142	2074–2150 ^b^	[10]	0.1
74	Ethyl cinnamate	2154	2157 ^a,c,m^	[9,11,18]	6.8
75	*T*-Cadinol	2166	2136–2200 ^b^	[10]	0.7
76	Cinnamyl acetate	2170	2174 ^a,c^	[9,11]	0.1
77	4-Ethyl phenol	2178	2195 ^a^, 2196 ^c^	[9,11]	1.1
78	Germacrene-D-4-ol	2194	2202 ^c^	[11]	0.1
79	T-Muurolol	2201	2153–2209 ^b^	[10]	tr
80	Torreyol	2212	2219 ^a,c^ 2135–2219 ^b^	[9,10,11]	1.6
81	Elemicine	2230	2245 ^c^	[11]	tr
82	Isobutyl cinnamate	2272	2282 ^c^	[11]	0.1
83	Cinnamyl alcohol	2290	2308 ^a,c^	[9,11]	0.4
84	(2*E*,6*Z*)-Farnesol	2303	2219–2342 ^b^	[10]	tr
85	Chavicol	2334	2353 ^c^ 2320–2358 ^b^	[10,11]	0.1
86	(2*Z*,6*E*)-Farnesol	2336	2340–2370 ^b^	[10]	tr
87	Butyl cinnamate	2342	-	-	tr
88	Eudesma-4(15),7-dien-1β-ol	2354	2369 ^c^ 2351–2402 ^b^	[10,11]	tr
89	4-Vinylphenol	2372	2398 ^n^	[19]	tr
90	Amyl cinnamate	2448	-	-	0.1
Total					98.9

LRIc: Relative retention indices calculated against n-alkanes (C7-C40) on a polar column; LRIr: Literature retention indices obtained from the published references ^a^ [9]; ^b^ [10]; ^c^ [11]; ^d^ [12]; ^e^ [13]; ^f^ [14]; ^g^ [15]; ^h^ [16]; ^k^ [17]; ^m^ [18]; ^n^ [19] for polar column values; %: Relative percentage calculated from FID data; tr: trace (<0.1).

**Table 2:** MIC, MBC, and MBC/MIC ratios of *L. orientalis* essential oil against tested bacterial isolates.

No	Bacteria and Their Source	MIC (µg/mL)	MBC (µg/mL)	MBC/MIC Ratio	Interpretation
1	*E. coli* (ATCC 25922)	15.62	31.25	2.00	Bactericidal
2	MDR *E. coli* (Animal-derived)	31.25	125.00	4.00	Bactericidal
3	*E. coli* (Human-derived)	15.62	31.25	2.00	Bactericidal
4	*E. coli* (Animal-derived)	15.62	31.25	2.00	Bactericidal
5	*S. aureus* (ATCC 6538)	31.25	125.00	4.00	Bactericidal
6	MRSA (ATCC 43300)	62.50	125.00	2.00	Bactericidal
7	MDR *S. aureus* (Animal-derived)	31.25	62.50	2.00	Bactericidal
8	*S. aureus* (Human-derived)	62.50	250.00	4.00	Bactericidal
9	*S. aureus* (Animal-derived)	31.25	125.00	4.00	Bactericidal
10	*E. faecalis* (ATCC 29212)	62.50	125.00	2.00	Bactericidal
11	*E. faecium* (ATCC 6057)	31.25	125.00	4.00	Bactericidal
12	*K. pneumoniae* (Animal-derived)	62.50	125.00	2.00	Bactericidal
13	*K. pneumoniae* (Human-derived)	62.50	125.00	2.00	Bactericidal
14	*A. baumannii* (Human-derived)	ND	ND	ND	ND
15	*A. baumannii* (Human-derived)	ND	ND	ND	ND
16	*L. monocytogenes* (ATCC 7644)	31.25	62.50	2.00	Bactericidal
17	*S*. Typhimurium (NCTC 12416)	31.25	62.50	2.00	Bactericidal
18	*P. aeruginosa* (Human-derived)	125.00	250.00	2.00	Bactericidal
19	MDR *P. aeruginosa* (Animal-derived)	250.00	ND	ND	ND
20	*M. haemolytica* (Animal-derived)	62.50	125.00	2.00	Bactericidal
21	*M. haemolytica* (Animal-derived)	31.25	62.50	2.00	Bactericidal
22	*B. melitensis* (16M) (Animal-derived)	15.62	31.25	2.00	Bactericidal
23	*B. melitensis* (Animal-derived)	15.62	31.25	2.00	Bactericidal
24	*C. pseudotuberculosis* (Animal-derived)	31.25	62.50	2.00	Bactericidal

Note: Antibacterial activity was classified based on the MBC/MIC ratio: ≤4, bactericidal; >4, bacteriostatic. ND: Not detected.

**Table 3:** Inhibition zone diameters (mm) of *L. orientalis* essential oil against tested bacterial strains in the disk diffusion test (zones ≤ 7 mm considered to show no antibacterial activity).

				Concentration of *L. orientalis* Essential Oil (µg/mL)						Antibiotics	
Bacteria and Their Source		250	125	62.5	31.25	15.62	7.81	3.9	1.95	CN	S
		Inhibition Zone Diameter (mm)									
1	*E. coli* (ATCC 25922)	10.4 ± 0.2	9.9 ± 0.3	9.6 ± 0.8	8.5 ± 0.3	7.7 ± 0.4	7.1 ± 0.1	6.1 ± 0.3	6.0 ± 0.2	20.7 ± 0.1	16.2 ± 0.2
2	MDR *E. coli* (Animal-derived)	8.8 ± 0.3	8.2 ± 0.4	7.9 ± 0.4	7.7 ± 0.2	7.5 ± 0.1	7.3 ± 0.2	6.8 ± 0.3	6.2 ± 0.2	11.8 ± 0.2	12.6 ± 0.3
3	*E. coli* (Human-derived)	7.2 ± 0.3	6.9 ± 0.4	6.3 ± 0.2	6.1 ± 0.1	6.0 ± 0.0	6.0 ± 0.0	6.0 ± 0.0	6.1 ± 0.0	15.6 ± 0.3	12.9 ± 0.2
4	*E. coli* (Animal-derived)	11.3 ± 0.2	10.9 ± 0.3	10.7 ± 0.2	9.9 ± 0.4	8.4 ± 0.2	8.2 ± 0.1	7.3 ± 0.3	6.2 ± 0.2	11.2 ± 0.4	12.1 ± 0.2
5	*S. aureus* (ATCC 6538)	7.5 ± 0.5	7.3 ± 0.5	7.1 ± 0.2	6.7 ± 0.2	6.5 ± 0.1	6.2 ± 0.4	6.1 ± 0.3	6.1 ± 0.2	27.6 ± 0.2	21.3 ± 0.4
6	MRSA (ATCC 43300)	7.1 ± 0.4	6.9 ± 0.2	6.5 ± 0.5	6.3 ± 0.7	6.2 ± 0.5	6.1 ± 0.2	6.1 ± 0.0	6.1 ± 0.0	20.4 ± 0.4	13.2 ± 0.1
7	MDR *S. aureus* (Animal-derived)	6.8 ± 0.5	6.6 ± 0.1	6.4 ± 0.3	6.1 ± 0.3	6.1 ± 0.0	6.1 ± 0.0	6.1 ± 0.0	6.1 ± 0.0	10.0 ± 0.1	6.1 ± 0.2
8	*S. aureus* (Human-derived)	7.6 ± 0.3	7.5 ± 0.3	7.3 ± 0.5	7.2 ± 0.3	6.9 ± 0.2	6.5 ± 0.5	6.4 ± 0.2	6.1 ± 0.3	19.3 ± 0.2	9.1 ± 0.2
9	*S. aureus* (Animal-derived)	14.3 ± 0.1	13.4 ± 0.2	12.1 ± 0.2	11.5 ± 0.2	10.7 ± 0.2	8.2 ± 0.1	7.1 ± 0.2	6.6 ± 0.5	24.4 ± 0.1	33.0 ± 0.1
10	*E. faecalis* (ATCC 29212)	6.8 ± 0.3	6.8 ± 0.3	6.7 ± 0.3	6.5 ± 0.6	6.2 ± 0.3	6.1 ± 0.5	6.0 ± 0.2	6.0 ± 0.1	26.3 ± 0.1	18.3 ± 0.2
11	*E. faecium* (ATCC 6057)	8.3 ± 0.2	8.2 ± 0.7	8.1 ± 0.5	8.1 ± 0.4	7.8 ± 0.6	7.4 ± 0.2	7.0 ± 0.0	6.4 ± 0.2	14.1 ± 01	23.0 ± 0.1
12	*K. pneumoniae* (Animal-derived)	6.8 ± 0.2	6.4 ± 0.1	6.1 ± 0.2	6.0 ± 0.0	6.0 ± 0.0	6.0 ± 0.0	6.0 ± 0.0	6.0 ± 0.0	15.2 ± 0.3	11.1 ± 0.3
13	*K. pneumoniae* (Human-derived)	7.1 ± 0.3	7.0 ± 0.2	6.7 ± 0.4	6.3 ± 0.1	6.1 ± 0.4	6.0 ± 0.0	6.0 ± 0.0	6.0 ± 0.0	17.7 ± 0.2	8.3 ± 0.4
14	*A. baumannii* (Human-derived)	8.1 ± 0.3	8.0 ± 0.2	7.6 ± 0.2	7.2 ± 0.2	6.2 ± 0.1	6.1 ± 0.2	6.0 ± 0.0	6.0 ± 0.0	25.6 ± 0.1	20.2 ± 0.2
15	*A. baumannii* (Human-derived)	7.2 ± 0.2	7.0 ± 0.4	6.8 ± 0.3	6.5 ± 0.1	6.0 ± 0.0	6.0 ± 0.0	6.0 ± 0.0	6.0 ± 0.0	18.3 ± 0.2	9.7 ± 0.2
16	*L. monocytogenes* (ATCC 7644)	8.3 ± 0.3	8.1 ± 0.4	7.7 ± 0.4	7.5 ± 0.2	7.3 ± 0.2	7.0 ± 0.6	6.6 ± 0.3	6.1 ± 0.2	15.8 ± 0.2	8.1 ± 0.3
17	*S*. Typhimurium (NCTC 12416)	8.7 ± 0.4	8.4 ± 0.1	7.7 ± 0.4	7.3 ± 0.2	6.8 ± 0.5	6.2 ± 0.2	6.1 ± 0.1	6.0 ± 0.1	21.5 ± 0.2	10.3 ± 0.1
18	*P. aeruginosa* (Human-derived)	7.8 ± 0.4	7.4 ± 0.6	7.2 ± 0.4	6.9 ± 0.2	6.5 ± 0.5	7.3 ± 0.2	7.0 ± 0.0	6.2 ± 0.4	20.6 ± 0.2	17.1 ± 0.2
19	MDR *P. aeruginosa* (Animal-derived)	6.9 ± 0.2	6.5 ± 0.4	6.3 ± 0.5	6.0 ± 0.1	6.0 ± 0.1	6.0 ± 0.1	6.0 ± 0.1	6.0 ± 0.1	15.4 ± 0.2	20.9 ± 0.3
20	*M. haemolytica* (Animal-derived)	9.7 ± 0.4	9.5 ± 0.1	9.2 ± 0.6	8.6 ± 0.3	8.2 ± 0.5	6.7 ± 0.5	6.4 ± 0.2	6.1 ± 0.2	28.7 ± 0.1	31 ± 0.2
21	*M. haemolytica* (Animal-derived)	8.7 ± 0.6	8.3 ± 0.5	7.7 ± 0.6	7.2 ± 0.1	6.2 ± 0.2	6.1 ± 0.1	6.0 ± 0.1	6.0 ± 0.1	22.2 ± 0.1	25.8 ± 0.1
22	*B. melitensis* (16M) (Animal-derived)	13.3 ± 0.2	13.8 ± 0.4	13.1 ± 0.2	12.8 ± 0.1	10.4 ± 0.3	9.6 ± 0.2	8.7 ± 0.1	7.8 ± 0.2	24.3 ± 0.2	27.3 ± 0.2
23	*B. melitensis* (Animal-derived)	12.8 ± 0.2	12.2 ± 0.4	12.1 ± 0.2	11.7 ± 0.1	10.0 ± 0.2	9.7 ± 0.3	8.7 ± 0.2	7.9 ± 0.1	27.1 ± 0.1	31 ± 0.1
24	*C. pseudotuberculosis* (Animal-derived)	10.3 ± 0.1	10.01 ± 0.2	9.7 ± 0.4	9.4 ± 0.4	8.6 ± 0.5	7.7 ± 0.4	7.1 ± 0.2	7.0 ± 0.1	23.4 ± 0.2	18.1 ± 0.2

CN: Gentamicin (10 μg, Oxoid, Basingstoke, UK), S: Streptomycin (10 μg, Oxoid, Basingstoke, UK), MDR *E. coli:* Multi-drug resistant *E. coli*, MRSA: Methicillin-resistant *S. aureus*, MDR *S. aureus*: Multi-drug resistant *S. aureus*, MDR *P. aeruginosa*: Multi-drug resistant *P. aeruginosa*.

**Table 4:** Inhibition zone diameters (mm) of *L. orientalis* essential oil against tested bacterial strains in the agar well diffusion test (zones ≤ 6 mm considered to show no antibacterial activity).

				Concentration of *L. orientalis* Essential Oil (µg/mL)						Antibiotics	
Bacteria and Their Source		250	125	62.5	31.25	15.62	7.81	3.9	1.95	CN	S
		Inhibition Zone Diameter (mm)									
1	*E. coli* (ATCC 25922)	14.3 ± 0.2	12.3 ± 0.4	11.02 ± 0.4	11.01 ± 0.2	9.7 ± 0.5	8.5 ± 0.3	7.5 ± 0.2	6.2 ± 0.3	18.5 ± 0.2	17.1 ± 0.2
2	MDR *E. coli* (Animal-derived)	7.3 ± 0.2	7.1 ± 0.2	6.9 ± 0.4	6.7 ± 0.2	6.5 ± 0.3	6.3 ± 0.1	6.2 ± 0.1	6.1 ± 0.1	23.4 ± 0.1	22.7 ± 0.2
3	*E. coli* (Human-derived)	6.8 ± 05	6.8 ± 0.3	6.6 ± 0.2	6.0 ± 0.3	6.0 ± 0.2	6.0 ± 0.3	6.0 ± 0.0	6.0 ± 0.0	18.2 ± 0.3	20.8 ± 0.1
4	*E. coli* (Animal-derived)	12.3 ± 0.4	11.3 ± 0.1	11.3 ± 0.6	11.1 ± 0.2	10.7 ± 0.5	9.3 ± 0.2	8.6 ± 0.2	7.3 ± 0.2	29.1 ± 0.2	27.6 ± 0.2
5	*S. aureus* (ATCC 6538)	8.4 ± 0.2	8.1 ± 0.3	7.9 ± 0.2	7.7 ± 0.2	7.4 ± 0.1	7.2 ± 0.3	7.1.0 ± 0.1	7.0 ± 0.0	33.1 ± 0.1	25.5 ± 0.3
6	MRSA (ATCC 43300)	7.3 ± 0.2	7.1 ± 0.2	6.7 ± 0.3	6.0 ± 0.2	6.0 ± 0.1	6.0 ± 0.0	6.0 ± 0.0	6.0 ± 0.0	20.3 ± 0.2	23.5 ± 0.1
7	MDR *S. aureus* (Animal-derived)	6.8 ± 0.3	6.6 ± 0.2	6.1 ± 0.1	6.0 ± 0.2	6.0 ± 0.2	6.0 ± 0.2	6.0 ± 0.2	6.0 ± 0.2	27.3 ± 0.1	24.1 ± 0.2
8	*S. aureus* (Human-derived)	7.2 ± 0.2	7.1 ± 0.1	6.8 ± 0.2	6.6 ± 0.1	6.4 ± 0.3	6.3 ± 0.1	6.2 ± 0.2	6.1 ± 0.1	13.5 ± 0.2	8.8 ± 0.1
9	*S. aureus* (Animal-derived)	11.7 ± 0.2	11.4 ± 0.4	11.3 ± 0.2	10.1 ± 0.2	9.7 ± 0.2	7.3 ± 0.2	6.9 ± 0.3	6.3 ± 0.4	22.7 ± 0.3	19.9 ± 0.2
10	*E. faecalis* (ATCC 29212)	7.2 ± 0.2	7.0 ± 0.1	6.4 ± 0.3	6.0 ± 0.3	6.0 ± 0.1	6.0 ± 0.0	6.0 ± 0.0	6.0 ± 0.0	15.1 ± 0.2	8.7 ± 0.3
11	*E. faecium* (ATCC 6057)	7.4 ± 0.3	7.2 ± 0.2	6.8 ± 0.1	6.7 ± 0.2	6.5 ± 0.3	6.3 ± 0.1	6.0 ± 0.0	6.0 ± 0.0	26.6 ± 0.2	25.3 ± 0.2
12	*K. pneumoniae* (Animal-derived)	7.8 ± 0.2	7.1 ± 0.4	6.7 ± 0.5	6.0 ± 0.2	6.0 ± 0.2	6.0 ± 0.0	6.0 ± 0.0	6.0 ± 0.0	17.5 ± 0.3	23.1 ± 0.2
13	*K. pneumoniae* (Human-derived)	6.0 ± 0.0	6.0 ± 0.0	6.0 ± 0.0	6.0 ± 0.0	6.0 ± 0.0	6.0 ± 0.0	6.0 ± 0.0	6.0 ± 0.0	14.8 ± 0.2	18.1 ± 0.2
14	*A. baumannii* (Human-derived)	9.1 ± 0.2	8.8 ± 0.1	8.6 ± 0.3	8.2 ± 0.2	7.9 ± 0.4	7.5 ± 0.2	7.0 ± 0.2	6.8 ± 0.4	25.2 ± 0.2	25.9 ± 0.3
15	*A. baumannii* (Human-derived)	6.0 ± 0.0	6.0 ± 0.0	6.0 ± 0.0	6.0 ± 0.0	6.0 ± 0.0	6.0 ± 0.0	6.0 ± 0.0	6.0 ± 0.0	7.5 ± 0.2	12.2 ± 0.2
16	*L. monocytogenes* (ATCC 7644)	9.2 ± 0.2	9.1 ± 0.3	8.8 ± 0.0	8.6 ± 0.2	8.4 ± 0.3	7.8 ± 0.2	7.4 ± 0.2	7.1 ± 0.5	24.5 ± 0.2	21.2 ± 0.1
17	*S*. Typhimurium (NCTC 12416)	7.4 ± 0.2	7.2 ± 0.0	7.0 ± 0.3	6.8 ± 0.1	6.7 ± 0.1	6.4 ± 0.1	6.3 ± 0.0	6.1 ± 0.1	11.2 ± 0.2	8.1 ± 0.2
18	*P. aeruginosa* (Human-derived)	6.8 ± 0.4	6.7 ± 0.2	6.2 ± 0.3	6.0 ± 0.2	6.0 ± 0.1	6.0 ± 0.0	6.0 ± 0.0	6.0 ± 0.0	22.1 ± 0.2	15.7 ± 0.2
19	MDR *P. aeruginosa* (Animal-derived)	6.4 ± 0.2	6.0 ± 0.0	6.0 ± 0.0	6.0 ± 0.0	6.0 ± 0.0	6.0 ± 0.0	6.0 ± 0.0	6.0 ± 0.0	27.0 ± 0.0	23.6 ± 0.1
20	*M. haemolytica* (Animal-derived)	11.9 ± 0.3	11.6 ± 0.2	11.0 ± 0.1	11.1 ± 0.1	10.7 ± 0.2	9.7 ± 0.3	8.3 ± 0.2	7.8 ± 0.2	16.7 ± 0.2	15.0 ± 0.0
21	*M. haemolytica* (Animal-derived)	8.6 ± 0.2	8.4 ± 0.3	8.1 ± 0.0	7.9 ± 0.2	7.6 ± 0.2	7.4 ± 0.3	7.1 ± 0.2	6.8 ± 0.2	19.5 ± 0.1	16.2 ± 0.1
22	*B. melitensis* (16M) (Animal-derived)	16.4 ± 0.1	15.7 ± 0.2	15.5 ± 0.1	15.1 ± 0.5	14.6 ± 0.2	13.7 ± 0.2	10.4 ± 0.1	10.2 ± 0.1	25.2 ± 0.2	20.2 ± 0.4
23	*B. melitensis* (Animal-derived)	14.7 ± 0.3	13.9 ± 0.1	13.6 ± 0.2	12.8 ± 0.2	11.7 ± 0.3	9.7 ± 0.4	9.2 ± 0.4	8.9 ± 0.2	24.1 ± 0.1	19.7 ± 0.2
24	*C. pseudotuberculosis* (Animal-derived)	11.4 ± 0.2	10.9 ± 0.1	10.6 ± 0.3	10.1 ± 0.2	9.4 ± 0.5	8.4 ± 0.3	8.1 ± 0.6	7.4 ± 0.2	21.6 ± 0.2	25.2 ± 0.2

CN: Gentamicin sulfate (CAS no. 1405-41-0, Sigma-Aldrich, Seelze, Germany), S: Streptomycin sulfate (Cat. no. 2311004, Menarini Health and Pharmaceutical Industry Trade Inc. Istanbul, Türkiye), MDR *E. coli*: Multi-drug resistant *E. coli*, MRSA: Methicillin-resistant *S. aureus*, MDR *S. aureus*: Multi-drug resistant *S. aureus*, MDR *P. aeruginosa:* Multi-drug resistant *P. aeruginosa*.

**Table 5:** FICI values of *L. orientalis* essential oil combined with antibiotics against tested bacterial strains.

Bacteria	Antibiotics	FICI Values	Interaction
MDR *S. aureus*	Amoxicillin trihydrate	0.18	Synergy
	Erythromycin A dehydrate	7.45	Antagonism
*L. monocytogenes* (ATCC 7644)	Amoxicillin trihydrate	0.32	Synergy
	Erythromycin A dehydrate	0.39	Synergy
MDR *E. coli*	Gentamicin sulfate	2.76	Indifference
	Enrofloxacin	8.95	Antagonism
*S*. Typhimurium (NCTC 12416)	Gentamicin sulfate	0.66	Additive
	Enrofloxacin	0.21	Synergy
*B. melitensis* (Animal-derived)	Tetracycline hydrochloride	0.31	Synergy
	Gentamicin sulfate	0.41	Synergy

**Table 6:** Antibiofilm activity of *L. orientalis* essential oil against bacterial strains at strain-specific sub-MICs.

No	Bacteria and Their Source	MIC (µg/mL)	Sub-MIC (µg/mL)	OD_570_ Untreated Control	OD_570_ Sub-MIC Treatment	Biofilm Inhibition (%)
1	*E. coli* (ATCC 25922)	15.62	7.81	1.23	0.78	36.59
2	MDR *E. coli* (Animal-derived)	31.25	15.62	1.24	0.76	38.71
3	*E. coli* (Human-derived)	15.62	7.81	0.12	0.12	No biofilm formation detected
4	*E. coli* (Animal-derived)	15.62	7.81	1.23	0.71	42.28
5	*S. aureus* (ATCC 6538)	31.25	15.62	0.20	0.20	No biofilm formation detected
6	MRSA (ATCC 43300)	62.50	31.25	1.00	0.65	35.00
7	MDR *S. aureus* (Animal-derived)	31.25	15.62	1.00	0.72	28.00
8	*S. aureus* (Human-derived)	62.50	31.25	0.20	0.20	No biofilm formation detected
9	*S. aureus* (Animal-derived)	31.25	15.62	1.00	0.54	46.00
10	*E. faecalis* (ATCC 29212)	62.50	31.25	0.94	0.68	27.66
11	*E. faecium* (ATCC 6057)	31.25	15.62	0.90	0.55	38.89
12	*K. pneumoniae* (Animal-derived)	62.50	31.25	0.73	0.42	No biofilm formation detected
13	*K. pneumoniae* (Human-derived)	62.50	31.25	0.70	0.44	No biofilm formation detected
14	*A. baumannii* (Human-derived)	ND	ND	0.60	0.41	31.67
15	*A. baumannii* (Human-derived)	ND	ND	0.15	0.15	No biofilm formation detected
16	*L. monocytogenes* (ATCC 7644)	31.25	15.62	0.81	0.50	38.27
17	*S*. Typhimurium (NCTC 12416)	31.25	15.62	1.00	0.56	44.00
18	*P. aeruginosa* (Human-derived)	125.00	62.50	0.80	0.44	45.00
19	MDR *P. aeruginosa* (Animal-derived)	250.00	ND	0.70	0.57	18.57
20	*M. haemolytica* (Animal-derived)	62.50	31.25	0.93	0.44	52.69
21	*M. haemolytica* (Animal-derived)	31.25	15.62	0.90	0.45	50.00
22	*B. melitensis* (16M) (Animal-derived)	15.62	7.81	0.82	0.31	62.20
23	*B. melitensis* (Animal-derived)	15.62	7.81	0.80	0.33	58.75
24	*C. pseudotuberculosis* (Animal-derived)	31.25	15.62	0.15	0.15	No biofilm formation detected

## Data Availability

All data generated or analyzed in this study are included in the published article, and additional raw data can be obtained from the corresponding authors upon reasonable request.

## Abbreviations

The following abbreviations are used in this manuscript:

GC-MS	Gas chromatography-mass spectrometry
MDR *E. coli*	Multi-drug resistant *E. coli*
MRSA	Methicillin-resistant *S. aureus*
MDR *S. aureus*	Multi-drug resistant *S. aureus*
MDR *P. aeruginosa*	Multi-drug resistant *P. aeruginosa*
